# The Thermal and Mechanical Properties of Poly(ethylene-*co*-vinyl acetate) Random Copolymers (PEVA) and its Covalently Crosslinked Analogues (cPEVA)

**DOI:** 10.3390/polym11061055

**Published:** 2019-06-17

**Authors:** Ke Wang, Qibo Deng

**Affiliations:** 1 School of Materials Science and Engineering, University of Shanghai for Science and Technology, 516 Jungong Road, Shanghai 200093, China; wangk2017@usst.edu.cn; 2 Institute for New Energy Materials and Low-Carbon Technologies, School of Materials Science and Engineering, Tianjin University of Technology, Tianjin 300384, China; 3 Key Laboratory of Advanced Energy Materials Chemistry (Ministry of Education), College of Chemistry, Nankai University, Tianjin 300071, China

**Keywords:** random copolymers, mechanical property, thermal property, covalent crosslinking

## Abstract

The thermal and mechanical properties of poly(ethylene-*co*-vinyl acetate) random copolymers (PEVA) and its covalently crosslinked analogues (cPEVA) were controlled by the overall crystallinity of the polymer networks. The cPEVAs with different VA-content were synthesized by thermally-induced crosslinking of linear PEVA with dicumyl peroxide (DCP). This work was mainly concerned with the effect of vinyl acetate (VA) content on the crosslinking density, thermal and mechanical properties of PEVAs and cPEVAs, respectively. The chemical composition was analyzed by thermogravimetric analysis and ^1^H-NMR. The thermal and mechanical properties of PEVAs and cPEVAs have been studied through a series of conventional analytical methods, including gel content determination, different scanning calorimetry, thermogravimetric analysis, dynamic mechanical thermal analysis and traditional mechanical measurements. The experimental results show that the thermal and mechanical properties of PEVAs and cPEVAs increase with decreasing the VA-content. A broad melting transition with a Δ*T*_m_ in the range from 78 °C to 95 °C was observed for all polymer networks.

## 1. Introduction

Shape memory polymers (SMP) are materials which can be deformed and fixed in a temporary shape, from which they recover their original, permanent shape when being exposed to a certain external stimulus, such as heat, light, electricity or magnetic field [1,2,3,4]. The emerging field of SMP has attracted tremendous interest due to its various potential applications covering smart packaging [5], heat-shrink tubing, deployable structures and microdevices [6], intelligent drug releasing systems and medical devices for minimally invasive surgery [7,8,9,10,11,12,13], etc. In the last decade, shape-memory semi-crystalline polymers with covalent crosslinking, e.g., degradable SMPs containing poly(ε-caprolactone) (PCL) switching segments [12,14,15] or covalent crosslinked poly(ethylene-*co*-vinyl acetate) (cPEVA) using polyethylene (PE) switching segments [16], have been widely studied and become popular in biomedical application.

**Figure 1:**
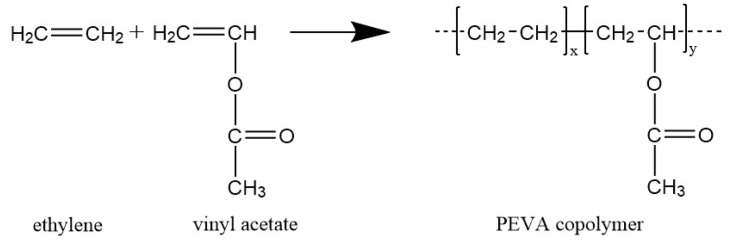
Chemical structure of monomers and poly(ethylene-*co*-vinyl acetate) random copolymers (PEVA).

Poly(ethylene-*co*-vinyl acetate) [17] is a random copolymer consisting of semi-crystalline polyethylene (PE) segments and amorphous poly(vinyl acetate) (PVA) segments. Figure 1 shows the chemical structure of monomers and PEVA copolymer. PEVA is a thermoplastic polymer extensively used in different fields, such as flexible packaging, footwear, hot met adhesives and cable sheathing. PEVA is also considered as a good candidate for biomedical application because of its ease of handling and processing, biocompatibility or drug delivery capability [18,19].

The physical and mechanical properties of PEVA are influenced by the crystallinity of copolymer [20,21,22,23], which can be adjusted by the variation of VA-content [17]. The formation of the crystalline structure is due to the organized arrangements of linear polyethylene chains in PEVA. As the increase of VA-content, the stereoregularity of polymer chains reduces, resulting in the decrease of the crystallinity of PE segments. Therefore, both the melting temperature and the storage modules of PEVAs can be reduced [17]. Brogly et al. [20] and Arsac et al. [21] reported that the melting temperature (*T*_m_) of different PEVAs decreases with the reduction of crystallinity, which results either from imperfection or variation of crystals. Sung et al. [17] also suggested that storage modulus of PEVAs declines with augment of VA-content at temperatures above *T*_g_ due to the reduction of crystallinity, which could reinforce the mechanical property of PEVAs.

On the other hand, crosslinking density also plays an important role in adjusting the material properties. In generally, the increase of crosslinking density can result in the increase of the material properties (modulus, hardness, resilience, and abrasion resistance) whereas the decrease of the elongation at break, heat build-up, and stress relaxation. Recent reports have shown that PEVAs can be crosslinked, either by the exposure of the polyethylene homopolymers to high-energy ray (e.g., electron beam or γ-ray), or by chemical crosslinking (e.g., peroxide or silane crosslinking) [17,24,25]. Li et al. [16] and Sung et al. [17] have proved the influence of crosslinking degree of cPEVA on its dynamic modulus around the melting temperature. Yao et al. [18] have also suggested that the thermal stability can be enhanced considerable by the crosslinking of PEVA. Crosslinked PEVA materials have been used for various fields such as photovoltaic modules, insulation materials, cables, damping mattress for railroad crossties, and shoe soles [26,27,28,29,30,31]. To improve its mechanical strength and thermal resistance properties, PEVA is generally cured by peroxide [32,33,34,35], such as dicumyl peroxide (DCP). The mechanism of the crosslinking is due to the formation of the radiacal of the DCP [36,37]. The DCP crosslinking reaction was carried out preferably on both VA-segments and PE-segments. How the –CH_2_ and –CH reacted and how much of them reacted can be characterized by Raman spectroscopy.

This study focuses to examine the effect of VA content on the properties of PEVA and cPEVA, which include the crosslink density, thermal and mechanical characteristics. These were examined through a series of conventional analytical methods, including gel content determination, different scanning calorimetry, thermogravimetric analysis, dynamic mechanical thermal analysis and mechanical measurements.

## 2. Materials and Methods

### 2.1. Materials

Poly(ethylene-*co*-vinyl acetate) copolymers (PEVAs) with various VA-contents were provided by DuPont company (Neu-Isenburg, Germany) with product names as: Elvax460 (18 wt% VA), Elvax3175LG (28 wt% VA), Elvax150 (32 wt% VA), and Elvax40w (40 wt% VA) and Polimeri Europa company (Milano, Italy) trade-name Greenflex ML30 (9 wt% VA). The linear copolymers were applied without further purification. Dicumyl peroxide (DCP) as crosslinking agent was purchased from Sigma-Aldrich (Taufkirchen, Germany) and used as received. The polyethylene homopolymer, which was nominated as PEAV00 in this work, were purchased from Sigma-Aldrich and used without further purification.

### 2.2. Synthesis of PEVA Copolymer Networks (cPEVA)

The polymer networks were prepared by a two-step process. In the first step, 100 g of the starting materials (PEVA) were mixed with 2 wt% of DCP via a twin-screw extruder (EuroPrismLab, Thermo Fisher Scientific, Waltham, MA, USA) at 110 °C and the rotating speed was 50 rpm. In the second step, the granulates of PEVA/DCP blends were prepared and then compression molded into 2D films with 1 mm thickness on a compression molding machine (type 200 E, Dr. Collin, Ebersberg, Germany). After a waiting time period of 5 min at 110 °C, the crosslinking reaction occurred by increasing the temperature to 200 °C while maintaining a pressure of 20 bar for 25 min.

### 2.3. Determination of Crosslink Density in cPEVAs

The conversion of the crosslinking reaction and the crosslinking was analysed by swelling tests, which are used to predict the number of crosslinks in a polymer network. As measure for the yield of the crosslinking reaction the gel content (*G*) was determined, while the crosslinking density is correlated to the volumetric degree of swelling (*Q*).

For determination of *G*, testing specimens of cPEVAs were immersed in about 20 mL toluene in glass vials and kept for two days at 50 °C in a thermo oven. Afterwards, the swollen samples were dried for four days at 50 °C under vacuum until a constant weight was reached (*m_d_*). The gel content (*G*) was calculated as the quotient of the mass of the dried samples after extraction (*m_d_*) to the mass of the original samples (*m_iso_*) [36] (Equation (1)). For each type of cPEVAs, three specimens were applied.

(1) $$G\%=\frac{m_{d}}{m_{iso}}\times 100\%$$

The degree of swelling (*Q*) was determined by immersing the extracted cPEVAs for two days in 20 mL toluene at 50 °C in a thermo oven and then measuring the mass of the swollen samples. The specific density of cPEVAs was measured by Ultrapycnometer 1000 (Quantachrome Corporation, Boynton Beach, FL, USA) at ambient temperature. The average values were calculated from 50 runs.

The degree of swelling *Q* was calculated as follows (Equation (2)):

(2) $$Q=\left[1+\rho_{2}\left(\frac{m_{sw}}{m_{d}\rho_{1}}-\frac{1}{\rho_{1}}\right)\right]\times 100\%$$

where *m_sw_* is the weight of samples in the swollen state, *m_d_* is the original weight of the sample in the dry state, and *ρ*_1_ and *ρ*_2_ are the specific densities of the swelling agent and polymer, respectively [37].

### 2.4. ^1^H-NMR Spectroscopy

The chemical composition of cPEVA was determined by ^1^H-NMR spectroscopy recorded at 25 °C on a 500 MHz Advance spectrometer (Bruker, Karlsruhe, Germany) using toluene-d^8^ as solvent and tetramethylsilane (TMS) as internal standard. Experiments were performed at 500 MHz (^1^H) resonance frequency with the spectral width of 10,000 Hz. The tested samples were prepared by swelling in toluene-d^8^ at 50 °C for 18 h prior to the measurements.

### 2.5. Thermogravimetric (TG) and Derivative Thermogravimetric (DTG)

TGA measurements were carried out on TGA 209 (NETZSCH, Selb, Germany) with temperature ranging from −25 °C to 700 °C and the heating rate was 20 °C·min^−1^. Derivative thermogravimetry, DTG, is the change in weight of the sample with respect to time, dα/dt. The area of the peaks is in proportion to the total change in the sample weight. The DTG curves were obtained by differentiation of the TG curves. Two distinct gradients were obtained from loss weight curve, which had been expect to be associated with the weight loss percentage of polyethylene segments and polyvinyl acetate segments of copolymer respectively. The sample ID of PEVAs was nominated according to the weight contents of VA determined by TGA.

### 2.6. Wide Angle X-ray Scattering (WAXS)

Wide angle X-ray scattering (WAXS) is an X-ray diffraction technique that can be used to determine the crystalline structure of polymers.

WAXS measurements were carried out with an X-ray diffraction system Bruker D8 Discover with a two-dimensional detector from Bruker AXS (Karlsruhe, Germany). The X-ray generator, producing copper K-alpha radiation with a wavelength of 0.154 nm, was operated at a voltage of 40 kV and a current of 40 mA. A graphite monochromator and a pinhole collimator with an opening of 0.8 mm defined the optical and geometrical properties of the beam. Samples were illuminated for 60 s in transmission geometry and the diffraction images of were collected at a sample-to-detector distance of 15 cm. The measurements were performed on the room temperature and the diffraction images were recorded from 8° to 42° of the scattering angles 2*θ*. The two-dimensional diffraction images were integrated to obtain plots with intensity versus diffraction angle. These profiles were analyzed by using the Bruker software TOPAS to determine the degree of crystallinity (DOC), which is the ratio of the area of crystalline peaks to the total area below the diffraction curve (area of crystalline peaks plus area of the amorphous halo).

### 2.7. Differential Scanning Calorimetry (DSC)

DSC measurements were performed on DSC 204 Phoenix (NETZSCH, Selb, Germany) including a heating-cooling-heating cycle. The first heating process was performed from ambient temperature to 200 °C with a heating rate of 20 °C·min^−1^ followed by cooling to −100 °C at varied cooling rate (100 °C·min^−1^, 50 °C·min^−1^, 20 °C·min^−1^, 10 °C·min^−1^, 5 °C·min^−1^ and 1 °C·min^−1^) for determination of the temperature, where crystallization occurred. The melting temperature and the glass transition temperature of cPEVAs were obtained from the second heating run from −100 °C to 200 °C. Furthremore, the crystallinity index (*χ_c_*) of PE segments in cPEVAs can be calculated from DSC exothermic curves according to Equation (3) [37]:

*χ_c_* = Δ*H_m_*/Δ*H*_100_ (3)

where Δ*H_m_* is the integrated melting enthalpy, representing the area of the melting peak [38], and Δ*H*_100_ is the specific melting enthalpy for a 100% crystalline PE segment (287.3 J·g^−1^) [39,40,41]

### 2.8. Dynamic Mechanical Analysis (DMA) at Varied Temperatures

DMTA measurements were carried out on GABO Eplexor 25 N (Gabo, Ahlden, Germany) equipped with a 25 N load cell using type DIN EN ISO 527-2/1BB test specimen, of which the width was 2 mm, the length is 20 mm and thickness is around 1 mm. The static load was 10 N, and the dynamic load was 3 N. The applied oscillation frequency was 10 Hz. All of the measurements were performed in temperature sweep mode from −140 °C to 110 °C with a constant heating rate of 2 °C·min^−1^. *T*_δ,max_ was determined at the peak maximum of tan *δ*-temperature curve.

### 2.9. Mechanical Testing

Tensile tests were performed on a universal tensile tester Zwick 87 (Zwick, Ulm, Germany) using type DIN EN ISO 527-2/1BB test specimen (the width is 2 mm, the length is 20 mm and thickness is around 1 mm) at a speed of 5 mm·min^−1^ at ambient temperature for the PEVAs and cPEVAs. For each sample, five measurements were carried out. The deformation of a material in a predefined profile was measured and stress-strain curves of samples were given out by software. Tensile strength (*σ*), Young’s Modulus (*E*) and elongation at break (*ε*_b_) of the polymer network could be determined according to stress-strain curve. The Young’s modulus *E* is experimentally determined from the slope of stress-train curves generated by tensile tests at an initial strain of 0.02% to 0.5%.

## 3. Results and Discussion

### 3.1. Composition of PEVA and cPEVA

#### 3.1.1. Determination of VA-Content

**Figure 2:**
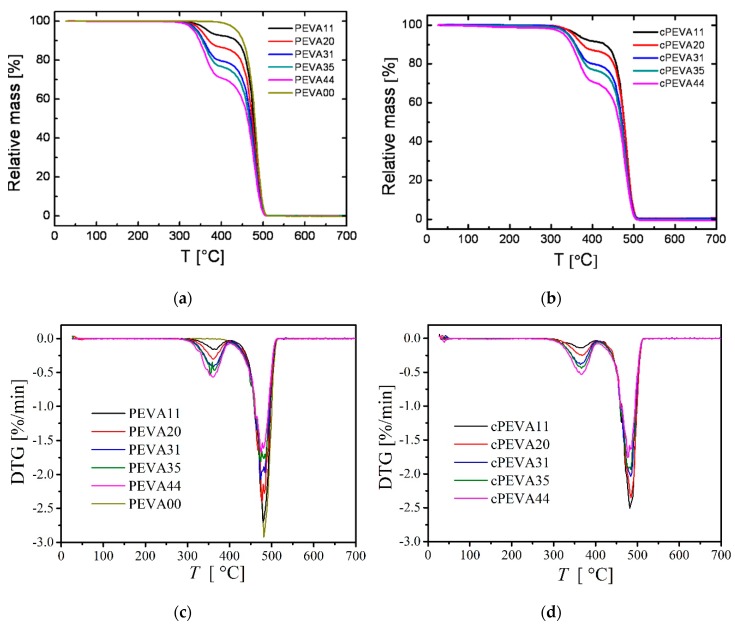
Thermal stability of polyethylene homopolymer (PEAV00), PEVAs (**a**) and cPEVAs (**b**) with varied vinyl acetate contents determined by TGA and derivative thermogravimetric (DTG) of polyethylene homopolymer (PEAV00), PEVAs (**c**) and cPEVAs (**d**) with temperature from 25 °C to 700 °C, at a heating rate of 20 °C·min^−1^.

The chemical composition of in this work was nominated PEVAs and cPEVAs was investigated by thermogravimetric (TG) and derivative thermogravimetric (DTG), and ^1^H-NMR spectroscopy. The composition of PEVA and cPEVAs was explored by TGA and DTG analysis. TGA has the capability of measuring the mass loss and derivative, whereas DTG has the capability of measuring the temperature difference as a function of temperature. The obtained TGA and DTA curves vs. temperature for PEVAs and cPEVAs are shown in Figure 2. Here two decomposition steps of PEVA and cPEVAs [42] were observed, which are related to the generation of volatile products. The first peak of the DTG curves occurs in the temperature range from 300 °C to 410 °C with weight loss around 11% to 44%, which can be attributed to the deacetylation of VA segments, while at higher temperature range around 420–510 °C, the second peak appears for the decomposition of the residuals induced by the cracking of C-C bond along main chains.

Thus, the composition of cPEVA could be determined according to the weight loss of acetic acid groups obtained in the temperature range from 300 °C to 410 °C, where the weight content of VA segment was calculated by Equation (4).

(4) $$\text{VA-content wt}\%=\frac{w_{1}\cdot \left(M_{w}^{VA}/M_{w}^{-COOCH_{3}}\right)}{w}\times 100\%$$

where *w*_1_ is the weight loss attributed to the deacetylation of VA segments in the temperature range from 300 °C to 410 °C and *w* is total weight of PEVA and cPEVA samples. *M_w_* is the molar mass of VA and acetic acid groups. The results are listed in Table 1, which are closed to parameters provided by DuPont Company. The sample ID of cPEVA in this work was nominated according to the weight content of vinyl acetate (VA) determined by TGA and DTA. Slight variation of VA-content was observed for some PEVAs and its covalently crosslinked analogues, which might be attributed to the experimental error in the range of 1–2 wt%.

**Figure 3:**
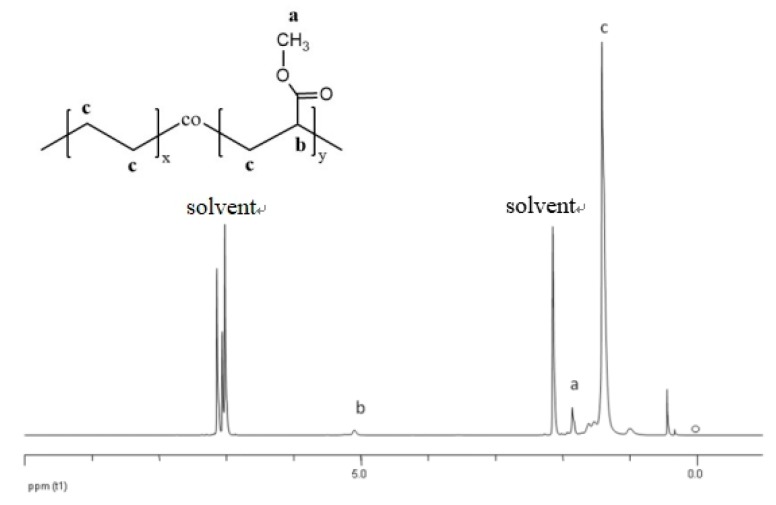
^1^H-NMR spectrum of cPEVA31 with assigned chemical shifts. a: –CH_3_ (protons from methyl group, δ = 1.8 ppm); b: –CH– (protons from methine group, δ = 5.0 ppm); c: –CH_2_– (linear methylene protons, δ = 1.3–1.6 ppm). The solvent (toluene-d^8^) appears at δ = 2.2 and 7.1 ppm.

Complementary to TGA ^1^H-NMR measurements of cPEVA swollen in toluene-d^8^ were applied for determination of the composition. A typical ^1^H-NMR spectrum obtained for cPEVA31 is displayed in Figure 3, with assigned chemical shifts; a: –CH_3_ (protons from methyl group, δ = 1.8 ppm); b: –CH– (protons from methine group, δ = 5.0 ppm); c: –CH_2_– (linear methylene protons, δ = 1.3–1.6 ppm). The solvent (toluene-d^8^) appears at δ = 2.2 and 7.1 ppm.

The weight content of VA segment was calculated according to Equation (5).

(5) $$\text{VA-content wt}\%=\frac{x\cdot M_{w}^{VA}}{x\cdot M_{w}^{VA}+y\cdot M_{w}^{E}}\times 100\%$$

*M_w_* is the molar mass of VA and ethylene monomers; *x* and *y* are the molar ratio between VA and ethylene repeating units and were calculated according to the intensity of signals b and c, where c = 4*y* + 2b and b = *x*. The cPEVA composition determined by ^1^H-NMR analysis is almost in agreement with the results obtained by TGA, where the VA-content increased from about 10 wt% for cPEVA11 to 41 wt% for cPEVA44.

#### 3.1.2. Degree of Crystallinity

The crystallinity index (*χ*_c_) of PE segments in PEVA or cPEVA was calculated from the Δ*H*_m_ values determined by DSC, according to Equation (3). When increasing VA-content, the value of *χ*_c_ decreases systematically from 33.7% for PEVA11 to 3.7% for PEVA44, and from 30% for cPEVA11 to 5.2% for cPEVA44, respectively. The results are shown in Table 1.

**Figure 4:**
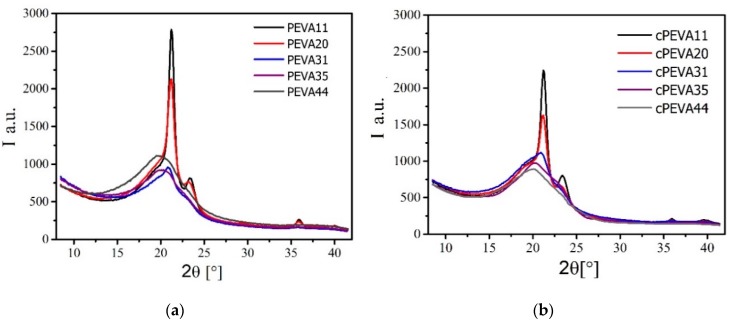
Wide-angle X-ray scattering (WAXS) patterns of PEVAs (**a**) and cPEVAs (**b**) with various VA-contents.

Complementary to *χ*_c_ calculated from the DSC results, wide-angle X-ray scattering (WAXS) was used to determine the degree of crystallinity (DOC). The WAXS patterns of the PEVAs and cPEVAs with different VA-content are shown in Figure 4. The X-ray patterns for the PEVAs and cPEVAs become broader with the increase of the VA-content, indicating the decrease of the crystallinity of PEVAs and cPEVAs with the increase of VA-content. This is in agreement with *χ*_c_ results as well as the previous reports about covalently crosslinked PEVA [17]. It is caused by the reduction of the stereogularity of polyethylene main chain due to inducing the polarized acetate groups [17].

The degrees of crystallinity (DOC) of PEVAs and cPEVAs calculated by WAXS software are listed in Table 1 as well. Comparing the degree of crystalline of cPEVAs with PEVAs, it shows that the degree of crystalline decreases by the crosslinking of PEVA. It could because that the segments with suitable length for crystallization is reduced by formation of crosslinking and the networks restrain the arrangements of macromolecular chains in crystal lattice, which results in smaller, imperfect crystals [18]. However, the deviation of results between *χ*_c_ and DOC could be attributed to the integrated melting enthalpy Δ*H*_m_ which partly overlapped with the glass transition process and was affected by the calculated area of melting peak.

#### 3.1.3. Crosslinking Density

A two-step preparation method was applied to ensure that the initiation of the crosslinking process in step two and no decomposition of DCP happened during the melt blending process. This was confirmed by the preliminary solubility experiments, where most of the blends (PEVA + 2 wt% DCP) were completely dissolved in toluene. Only PEVA11/DCP blend was an exceptional sample, which was not totally soluble, therefore no swelling experiments were conducted with cPEVA11.

Gel content *G* of cPEVA were calculated according to Equation (1) and listed in Table 1. The gel content *G* of all cPEVAs measured in toluene is around 95%, which indicates a high conversion of crosslinking reaction [16,43]. The volumetric degree of swelling *Q*, which can be taken as a measure for the crosslinking density of a polymer network, was found to be almost independent from the VA-content in the range of 615% to 695%. The volumetric degree of swelling *Q* calculated by Equation (2) for the different cPEVA samples are shown in Table 1. The crosslinking of PEVA is a chain reaction [44] so only small weight percent of DCP of the total amount are sufficient to generate a well crosslinked network. From the previous study [45], 2 wt% dicumyl peroxide should be enough for crosslinking reaction. In principle, the higher crosslinking density, the more crosslinking points exist, which result in a less amount of solvent molecules penetrating into the polymer networks thus lead to a decline of swelling degree. So it is easily to infer that a small swelling degree simply represents small compartments of polymer networks with more crosslinking points, and vice versa [1]. Polyethylene, as well as polyvinyl acetate, can be crosslinked by DCP [46,47], which implies that the crosslinking of PEVA by DCP could happen both in PE segments and VA segments. Therefore, the crosslinking points of polymer networks might be related to the ratio of PE and VA-content. However, according to the Kim et al. [44], the most of the reactive points in the crosslinking process of PEVA were on carbon atoms with substitute acetate groups in the main chain.

### 3.2. Thermal Properties

#### 3.2.1. DSC Analysis

The thermal behavior of PEVAs and cPEVAs with different VA-content and crosslink density has been studied by differential scanning calorimetry (DSC), which is applied to determine the thermodynamic properties such as phase transition (e.g., glass transition or melting transition) and changes in heat capacity of polymers during the heating or cooling procedures. All obtained data are listed in Table 2.

**Figure 5:**
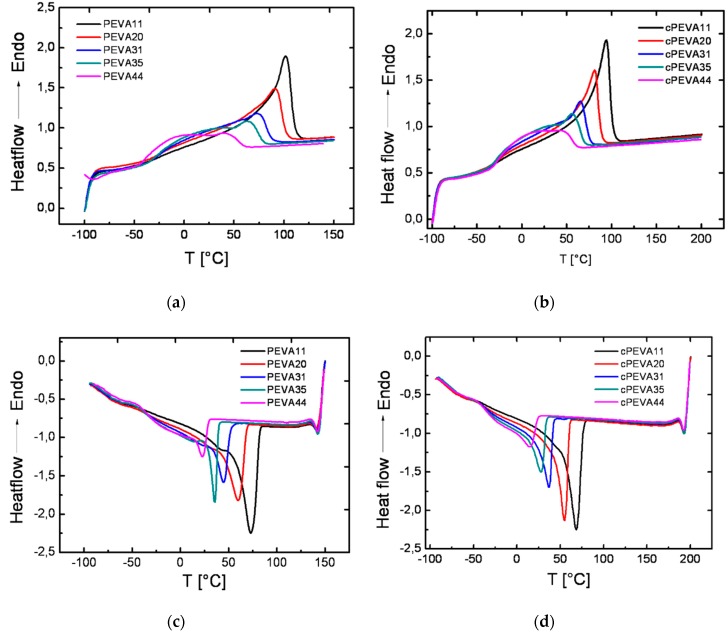
The second heating curves of DSC thermograms for PEVAs at a temperature range of −100 °C to 150 °C (**a**) and cPEVAs determined between −100 °C and 200 °C (**b**) with a heating rate of 20 C·min^−1^, where the melting temperature (*T*_m_) was obtained as the endothermic peak maximum in the heating curve and the glass transition temperature (*T*_g_) was shown as a step-wise transition in the thermograms, partly overlapped with the melting peak. The first cooling step of DSC thermograms for PEVAs with a temperature range of −100 °C to 150 °C (**c**) and cPEVAs determined in the temperature range between −100 °C and 200 °C (**d**) with a cooling rate of 20 C·min^−1^, where the crystallization temperature (*T*_c_) was determined as the peak maximum.

In Figure 5 the DSC heating and cooling curves (heat flow versus temperature) obtained in the second heating and first cooling run with a constant heating and cooling rate of 20 °C·min^−1^ are displayed, which were used for determination of melting temperature (*T*_m_), glass transition temperature (*T*_g_) and temperature of crystalline (*T*_c_).

A glass transition is a second-order endothermic transition, which appears as a step-wise transition in the second heating curves of DSC thermograms. For all tested PEVAs and cPEVAs, a glass transition with a *T*_g_ around −28 °C was observed, which was similar to the reported *T*_g_ at −25 °C by Reding et al. [48] and was found to be independent from the VA-content, as well as the crosslink density. The observed *T*_g_ of PEVA or cPEVA is related to the amorphous phase, consisting of rigid amorphous PE and amorphous PVA segments. No difference was observed between the *T*_g,_ of cPEVA and linear PEVA, indicating that the thermal properties were not affected by the crosslinking reaction.

A melting temperature (*T*_m_) is a first-order transition, which is normally observed as endothermic peak maximum in DSC heating curves. The *T*_m_ of PEVAs and cPEVAs decreased from 102 °C to 45 °C and from 94 °C to 41 °C, respectively, with increasing VA-content from 11 wt% to 44 wt%. This is because of the reduction of the regularity in PE semi-crystalline phases after incorporation of amorphous VA [17]. In addition, the crystallization behaviour was found to vary systematically for PEVAs and cPEVAs with different VA-content. Here *T*_c_ was observed at 24 °C for PEVA44 and at 73 °C for PEVA9, and at 14 °C for cPEVA44 and at 68 °C for cPEVA9, similar to the effect detected for *T*_m_ [17].

The melting temperature interval (Δ*T*_m_) ranging from *T*_m,onset_ to *T*_m,end_ was found to decrease from Δ*T*_m_ ≈ 108 °C for PEVA11 to Δ*T*_m_ ≈ 62 °C for PEVA44, and from Δ*T*_m_ ≈ 92 °C for cPEVA11 to Δ*T*_m_ ≈ 60 °C for cPEVA44, respectively. This observation can be explained by a broadened distribution of PE crystal size, which showed different *T*_m_ in PEVAs and cPEVAs because smaller and less imperfect crystallites generally exhibit a melting behaviour at lower temperatures [16]. The melting enthalpy (Δ*H*_m_), which reflects the amount of thermal energy absorbed for phase transition from semi-crystalline to liquid-like state, is represented by the area under the melting peak in the DSC curve. Δ*H*_m_ decreased significantly with increasing VA-content from 86 J·g^−1^ (cPEVA11) to 15 J·g^−1^ for cPEVA44, which might due to the hindrance caused by acetate side groups for the reduction of stereoregularity of main chain and thus results in the decrease of imperfection of the crystallites.

In addition it was observed that *T*_m_ as well as *T*_c_ of PEVAs is around 10 °C higher than its covalently crosslinked analogues cPEVAs, which can be explained by a reduction in PE segments length suitable for crystallization causing networks rearrangements of macromolecular chains in crystal lattice, which results in smaller, imperfect crystals [18].

**Figure 6:**
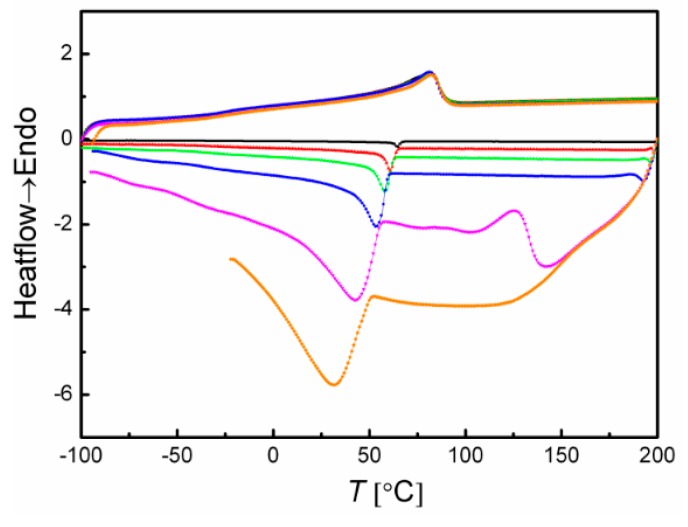
The second heating curves and the first cooling curves of DSC thermograms determined between −100 °C and 200 °C for cPEVA20D20 with the same heating rate of 20 °C·min^−1^ and different cooling rate of 1 °C·min^−1^ (black), 5 °C·min^−1^ (red), 10 °C·min^−1^ (green), 20 °C·min^−1^ (blue), 50 °C·min^−1^ (pink) and 100 °C·min^−1^ (orange).

For investigation of the influence of the cooling rate (*β_c_*) on the crystallization behaviour of cPEVA, additional DSC measurements were conducted for cPEVA20 where *β_c_* was varied from 1, 5, 10, 20, 50 to 100 °C·min^−1^, which was shown in Figure 6. The *T*_c_ values decreased from 65 °C to 31 °C with increasing *β_c_*, while *T*_m_ and Δ*H*_m_ were not influenced.

#### 3.2.2. DMA Analysis

**Figure 7:**
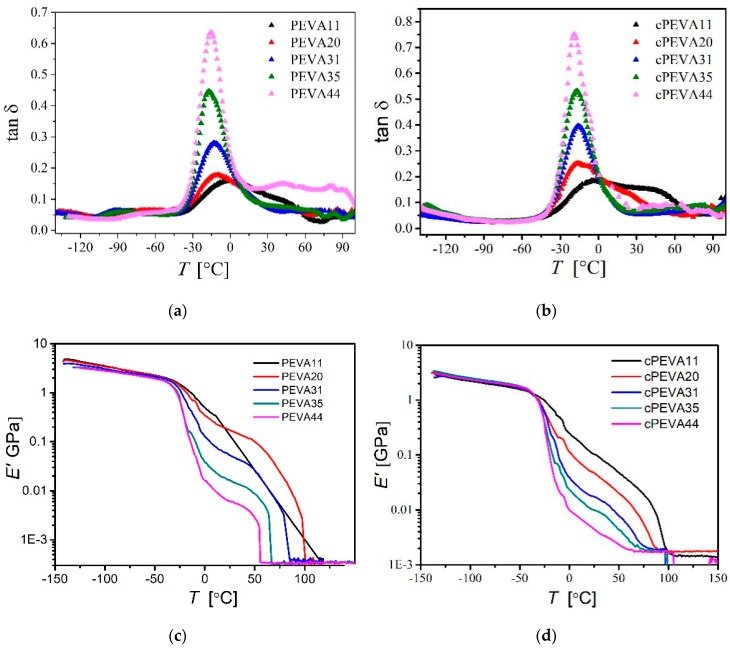
Dynamic mechanical analysis (DMA) at varied temperatures: (**a**) tan*δ*-temperature curves of PEVAs with different VA-contents. Two relaxation glass transition processes were observed for PE domains and copolymer chain. (**b**) tan*δ*-temperature curves of cPEVAs, where only one relaxation progress was observed as glass transition step of copolymer chain. (**c**) Storage modulus (*E*′)-temperature curves of PEVAs. (**d**) Storage modulus (*E*′)-temperature curves of cPEVAs.

In addition to DSC analysis, DMTA measurements at varied temperature were also conducted. DMTA studies the thermomechanical and damping properties of the materials, where the storage modulus (E′) and loss modulus (E″) can be determined, while the mechanical loss factor tan δ is calculated by the ratio of E″ to E′. The loss tangent tan δ and storage modulus E′ of the samples determined by DMTA were shown in Figure 7 and summarized in Table 2 for different PEVAs and cPEVAs. The temperature of maximum peak of tan*δ* is related to the glass transition temperature (*T*_δ,max_) of PEVAs and cPEVAs.

In the literature there were two glass transitions reported for PE determined by torsion braid analysis [49], a *T*_g_ at −83 °C associated to the truly amorphous linear PE and a second transition at −13 °C corresponding to amorphous region constrained by crystalline region, which refers to the rigid amorphous PE. In Figure 7a, for linear PEVAs we observed (*T*_g,PE_), at around −90 °C, could be associated with the motion of CH_2_ groups in sequence, involving only a few ethylene units, either three or five [48,50]. We assume that the original phase morphology of amorphous PE was disturbed by crosslinking reaction and therefore the transition was weakened and shifted to lower temperatures. The position of tan*δ* peaks depends on the frequency.

A second relaxation in temperature range from −50 °C to 30 °C with a pronounced peak maximum (T_δ_,_max_) in the temperature range of −32 °C to −3 °C for PEVA and −18 °C to −5°C for cPEVA, which is related to the glass transition of amorphous phase in PEVAs or cPEVAs, consisting of rigid amorphous PE and amorphous PVA segments [51]. *T*_δ,max_ was found to decrease with increase of VA-content. The determined thermal transitions of PEVA are in good agreement with the previously reported values for PEVA [50,52]. Reding et al. [48] reported that a glass transition of PEVA was at −25 °C, which was a characteristic of the motion of isolated –CH_2_–CHCOOCH_3_–CH_2_– groups. They suggested that the exact temperature of the transition depending on the nature of the acetate group (–COOCH_3_).

The intensity of tan δ peak was found to increase with decreasing VA-content, which can be used for quantification of the VA-content as reported by Lee et al. [53]. From Figure 7b, it was found that *T_δ_*, _max_ peak of cPEVAs with higher VA-contents (i.e., cPEVA11 and cPEVA20) was significantly broadened, which were attribute to the introduced crosslinks preferably located in the amorphous VA domains. It was observed that the *T*_δ,max_ have a small change by the crosslinking reaction, demonstrating that the crosslinking of PEVAs did not significantly affect the thermal properties, which were purely dependent on the chemical composition, i.e., the VA-content.

The schematics of the storage modulus (*E′*) depending on the temperature for different PEVAs and cPEVAs were shown in Figure 7c,d respectively. *E’* measures the rigidity of the specimens. As for semi-crystalline polymer, *E’* can be influenced by crystallinity. The increase of the crystalline phase can enhance *E’* of polymer due to the order-arrangement of molecular chains. From Figure 7d, the storage modulus of cPEVAs below −30 °C is shown to be nearly the same, while above −30 °C it decreases with the increase of the VA-content related to the reduction of crystallinity. For each cPEVA, *E’* degrease with the increase of temperature above −30 °C, which is due to the increase of mobile chains in the network. The melting temperature (*T*_m_) of PEVAs can also be obtained by an inflection point of *E′*-*T* curve, where is a linear change of the storage modulus from the rubbery state (high modulus) at lower temperatures to the liquid state (low modulus) at high temperatures. *T*_m_ of each PEVA are summarized in Table 2 except for PEVA11, which was broken while reaching ambient temperature during the DMA measurement and cannot be used for analysis. *T*_m_ was determined from the inflection point of the *E*′-*T* curves above 50 °C, where *E*′ decreased.

**Figure 8:**
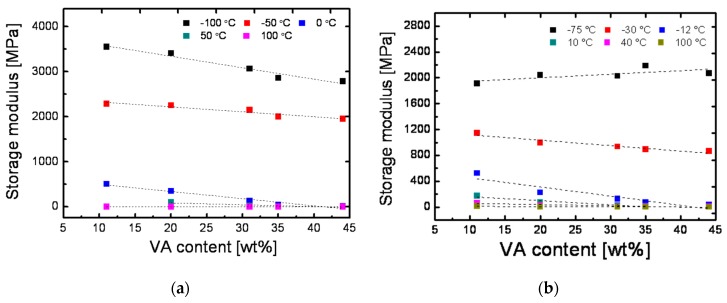
(**a**) Influence of VA-content on the storage modulus of PEVAs at various temperatures: −100 °C, −50 °C, 0 °C, 50 °C, and 100 °C. (**b**) Influence of VA-contents on the storage modulus of cPEVAs at various temperatures: −75 °C, −30 °C, −12 °C, 10 °C, 40 °C, and 80 °C.

In order to visualize the relationship between storage modulus *E’* and VA-content for cPEVAs at specific temperatures, the data of six different characteristic temperatures were analyzed according to the temperature range applied for cyclic thermomechanical testing. Each 6 temperature points are deployed to deduce the relationship between *E*′ and VA-content as displayed in Figure 8.

It is observed that slopes of the curves under the temperature of −75 °C, 40 °C and 100 °C are constant, which results from the temperature influences on the mobility of polymer chains. When the temperature is far below the *T*_δ,max_, the polymer chains are frozen and fixed, and the storage modulus becomes extremely high and cannot vary with the VA-content. With respect to the lowest curve in Figure 8b, polymer chains have sufficient energy to move at this high temperature, so that the restriction force as well as storage modulus are extremely low. As a result, the storage modulus still remains in constant and will be independent of VA-content for this temperature or even higher. In between, the storage modulus generally decreases with the increase of VA-content.

### 3.3. Mechanical Properties

**Figure 9:**
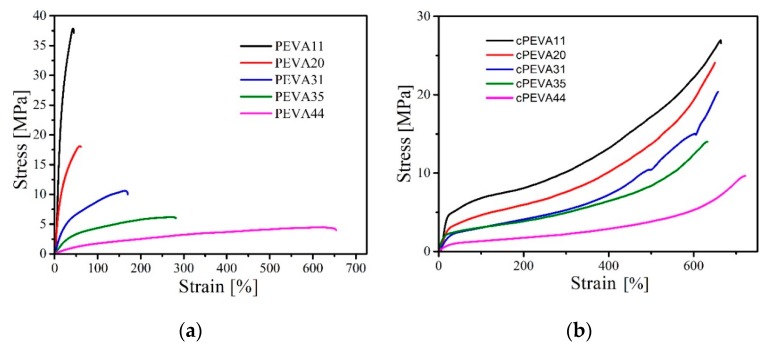
Stress–strain curves of PEVAs (**a**) and cPEVAs (**b**) determined by tensile tests at ambient temperature.

The mechanical properties of PEVAs and cPEVAs were investigated by uniaxial tensile tests at ambient temperature. Three characteristics were achieved from tensile test: Strength at break *σ*_b_, elongation at break *ε*_b_ and Young’s modulus (*E*), which are listed in Table 2. The strain-stress curves of PEVAs and cPEVAs are shown in Figure 9. The tensile strain-stress curves of cPEVAs at room temperature do not show an obvious yield point but a steady linear increase of stress with strain as it is typical for elastomers. As mentioned by Kim et al. [54], epoxies and perhaps some other thermosetting polymers appear to perform surprisingly well, at last in terms of predicted tensile strength and modulus. Gupta et al. [55] found the uniaxial tensile behavior of the epoxy depends on the glass transition temperature. The strain at break shows a significant increase when decreasing temperature approaching the glass transition, an effect previously reported in the literature [56,57]. From DMA results of cPEVAs, it could be seen that the glass transition temperature of all cPEVAs are below to the room temperature. Above *T*_g_, the molecular chains gave considerable flexibility, and the rubber-like elastic deformations are associated with changes in molecular conformation; the intermolecular forces are no longer operative and molecular architecture would not be expected to play an important part [55]. Thus, the cPEVAs show a Linear-yield-self strengthen-break, which is similar to the tensile behavior of crosslink network of hydroxyl-terminated polybutadiene binder [58].

The strength and stiffness of semi-crytalline networks can be tailored by crosslinking density and crystallinity. It can be seen that the stress at break *σ*_b_ decreased for both PEVAs and cPEVAs with increasing VA-content. This is might because the decrease of crystalline phase can lead to a reduction in mechanical stiffness of polymers. In contrast, the elongation at break *ε*_b_ was mainly depending on the crosslinking density and was similar for all cPEVAs. This is because cPEVAs have similar crosslinking density which can be seen from swelling tests results.

The Young’s modulus *E* is defined as the ratio of the uniaxial stress over the uniaxial in the range of the stress where Hooke’s Law holds. The *E* is experimentally determined from the slope of stress-train curves by tensile test, where the strain falls to the range of 0.02% to 0.5%. From Table 2, it can been seen that the values of Young’s modulus of PEVAs and cPEVAs decreased significantly with the increase of VA-content because of the reduction in degree of crystallinity.

Compared with PEVAs, *σ*_b_ and *ε*_b_ of corresponding cPEVAs increase by crosslinking, for example, PEVA31, *σ*_b_ increases from 8.5 MPa to 20.6 MPa, and *ε*_b_ from 101.1% to 675.6%. This can be mainly attributed to the existence of crosslinking structure of the cPEVAs that provides more stable skeleton under deformation. The *E* of cPEVAs is lower than that of the PEVAs at room temperature, which can be explained by a reduction of crystallinity after introduction of covalently crosslinks.

## 4. Summary and Outlook

cPEVAs were synthesized by introducing the crosslinks into the linear random poly(ethylene-*co*-vinyl acetate) copolymers (PEVA). High conversions were obtained according to G values above 94% for all cPEVAs. The effect of different VA-contents on degree of crystallinity, crosslink density, thermal and mechanical properties and has been systematically studied by a series of analytical methods, including ^1^H-NMR, TGA, DSC, DMTA, tensile tests, WAXS and the degree of swelling experiment.

Swelling experiment shows that gel content *G* of all cPEVAs is around 95%, which indicates a high conversion of crosslinking reaction. The crosslinking density is related to the ratio of PE segment and PVA segment in the network. The VA-contents of cPEVAs determined by ^1^H-NMR varied from 10 wt% for cPEVA11 to 41 wt% for cPEVA44, which agrees with the values determined from TGA curves. The results showed a two-step thermal degradation starting with the deacetylation of VA segments at around 300–410 °C and the cracking of C-C bond of ethylene main chains at 420–510 °C. The overall degree of crystallinity of cPEVAs determined by WAXS was found to decrease with increasing VA-content, which is in good agreement with the crystallinity index calculated from DSC data.

Thermal properties of PEVAs and cPEVAs were further analyzed by DSC and DMA. DSC shows the *T*_g_ of PEVAs and cPEVAs fall in the range of −25 °C to −30 °C and almost have no change with the different VA-content. *T*_m_, Δ*H*_m_ and *T*_c_ of both PEVAs and cPEVAs decreased with increase of the VA-content. Compared with PEVAs, DOC, the decrease on the *T*_m_ and *T_c_* of cPEVA suggests that the segments with a suitable length for crystallization are reduced by the introduction of crosslinking, which results in smaller, imperfect crystals. From DSC thermograms with varied cooling rate, the crystallization temperature *T*_c_ was shifted to lower values with increasing of the cooling rate, while *T*_m_ and Δ*H*_m_ were not influenced. DMA analysis shows two relaxation processes: the first weak relaxation process *T*_g,PE_ related to glass transitions of PEVA was found around −90 °C, which associated to truly amorphous PE segments; the second relaxation was observed in temperature range from −50 °C to 30 °C with a pronounced peak maximum (*T_δ_*,_max_) in the temperature range of −32 °C to −3 °C for PEVAs and −18 °C to −5°C for cPEVAs. This is related to a mixed amorphous phase of PEVA consisting rigid amorphous PE and amorphous PVA segments. The intensity of *T_δ_*,_max_ reveals that the amorphous phase in both PEVAs and cPEVAs is proportional to VA-content. The crosslinked network structures are more stable under deformation at around *T*_m_ compared to linear copolymers.

Mechanical properties were measured by tensile tests. The curves of cPEVAs show a steady linear increase of strain with stress without yield point like elastomers. The results of tensile tests demonstrate that, for cPEVAs, strength at break *σ*_b_, as well as Young’s modulus *E*, decrease with the increase of VA-content while the corresponding elongation at break *ε*_b_ was influenced by the crosslinking density.

In conclusion, the thermal and mechanical properties of the cPEVAs can be tailored by the VA-contents and degree of crystallinity. According to this comprehensive study and the results of the overall thermal and mechanical properties of PEVAs and cPEVAs, the engineering parameters (e.g., applied strain, strain rate and cooling rate, deformation, fixation and recovery temperature) can be accurately adjusted. The results are expected to be used for choosing appropriate cPEVAs, which is suitable for the shape memory effect test of cPEVAs with different degrees of crystallinity and crosslinking density.

## Figures and Tables

**Table 1:** Composition and crystallinity of PEVA and covalently crosslinked PEVA (cPEVA).

Sample ID ^a^	Product Name ^b^	VA-Content ^b^ [wt%]	VA-Content ^c^ [wt%]	VA-Content ^d^ [wt%]	*G*^e^ [%]	*Q*^f^ [%]	DOC ^g^ [%]	*χ*_c_^h^ [%]
**PEVA**								
PEVA11	Greenflex ML30	9	11	-	-	-	45.7 ± 0.7	33.7%
PEVA20	Elvax460	18	20	-	-	-	36.7 ± 0.8	24.8%
PEVA31	Elvax3175LG	28	31	-	-	-	27.6 ± 0.7	14.3%
PEVA35	Elvax150	32	35	-	-	-	13.1 ± 0.7	6.0%
PEVA44	Elvax40w	40	44	-	-	-	8.4 ± 0.8	3.7%
**cPEVA**								
cPEVA11			13	10	n.d *	n.d *	35.9 ± 0.1	30.0%
cPEVA20			21	18	96 ± 1	625 ± 15	27.6 ± 0.2	21.3%
cPEVA31			33	29	96 ± 1	695 ± 7	15.2 ± 0.1	16.4%
cPEVA35			37	33	94 ± 1	645 ± 8	7.8 ± 0.3	11.3%
cPEVA44			44	41	95 ± 1	615 ± 4	5.6 ± 0.4	5.2%

^a^ Sample ID. ^b^ The product names and the weight contents of vinyl acetate (VA) in the linear copolymers were provided by DuPont Company. ^c^ VA-contents determined by TGA. ^d^ VA-contents in cPEVAs were calculated according to ^1^H-NMR spectrum. ^e^ Gel content of cPEVAs calculated according to Equation (1). ^f^ Degree of swelling of cPEVAs calculated according to Equation (2). ^g^ Degree of crystallinity (DOC) measured by wide-angle X-ray scattering (WAXS). ^h^ Crystallinity (*χ*_c_) calculated based on the data of differential scanning calorimetry (DSC) according to Equation (3). * not determined.

**Table 2:** Thermal and mechanical properties of PEVA and cPEVA.

Sample ID	*T*_c_^a^ [°C]	*T*_m_^b^ [°C]	Δ*H_m_* ^c^ [J·g−1]	Δ*T* ^d^ [°C]	*T*_g_^e^ [°C]	*T*_g, PE_^f^ [°C]	*T*_δ,max_^g^ [°C]	*T*_m_^h^ [°C]	*E*^i^ [MPa]	*σ*_b_^j^ [MPa]	*ε*_b_^k^ [%]
PEVA11	73	102	96.9	108	−25	−84.5	−3	-	118.0 ± 70	18.9 ± 8	47 ± 6
PEVA20	65	91	71.2	104	−29	−65.4	−9	100	90.9 ± 17	13.2 ± 2.8	65 ± 4
PEVA31	45	73	41.0	91	−30	−92.4	−13	84	25.8 ± 5	8.5 ± 6	190 ± 8
PEVA35	36	63	17.2	65	−23	−87.8	−19	66	9.7 ± 1.2	4.1 ± 1	280 ± 30
PEVA44	24	45	10.6	62	−20	−84.5	−32	55	3.4 ± 0.2	3.2 ± 0.1	650 ± 36
cPEVA11	68	94	86.1	92	−26	n.o. ^l^	−5	-	33.7 ± 1.5	26.9 ± 5.3	664 ± 58
cPEVA20	55	81	61.1	79	−27	n.o.	−15	-	21.0 ± 5	24.1 ± 10	650 ± 30
cPEVA31	37	65	47.0	85	−30	n.o.	−16	-	10.4 ± 1.2	20.6 ± 3.4	657 ± 37
cPEVA35	29	56	32.6	73	−28	n.o.	−18	-	7.8 ± 0.7	14.0 ± 1.1	632 ± 18
cPEVA44	14	41	14.9	60	−26	n.o.	−18	-	2.9 ± 0.4	9.6 ± 0.7	721 ± 7

^a^*T*_c_ is the crystalline temperature determined in the first cooling curve of DSC measurement. ^b^ *T*_m_ is the melting temperature determined in the second heating curve of DSC measurement. ^c^ Δ*H_m_* is the integrated melting enthalpy calculated as the area under the curve of the melting process above the baseline in the second heating curve of DSC measurement. ^d^ Δ*T* is the interval of melting temperature from *T*_m,onset_ to *T*_m,end_ in the second heating curve of DSC measurement. ^e^ *T*_g_ is the glass transition temperature determined in the second heating curve of DSC measurement. ^f^ *T*_g, PE_ is the glass transition temperature of PE-domains determined from the first (from left to right) maximum tan*δ* peak in the tan*δ-*temperature curve of DMA. ^g^ *T*_δ,max_ is the second (from left to right) maximum tan*δ* peak in the tan*δ-*temperature curve of DMA measurements ^h^ *T_m_* is the melting temperature of PE crystal domains calculated as the inflection point from storage modulus (*E*′)-temperature curves of DMA measurements. ^i^ *E* is the Young’s modulus determined by tensile tests at ambient temperature. ^j^ *σ*_b_ is the stress at break. ^k^ *ε*_b_ is the elongation at break. ^l^ not observed.
